# Development of a diagnostic model to identify patients at high risk for cerebellopontine angle lesions

**DOI:** 10.1007/s00405-021-06778-6

**Published:** 2021-04-03

**Authors:** Mayke Hentschel, Maroeska Rovers, Stefan Steens, Gerjon Hannink, Henricus Kunst

**Affiliations:** 1 Department of Otolaryngology, Radboud Institute for Health Sciences, Radboud University Medical Center, Philips van Leydenlaan 15, P.O. Box 9101, 6500 HB Nijmegen, The Netherlands; 2 Department of Operating Rooms, Radboud Institute for Health Sciences, Radboud University Medical Center, Geert Grooteplein Zuid 10, Nijmegen, The Netherlands; 3 Department of Health Evidence, Radboud Institute for Health Sciences, Radboud University Medical Center, Geert Grooteplein 27, Nijmegen, The Netherlands; 4 Department of Radiology and Nuclear Medicine, Radboud University Medical Center, Geert Grooteplein Zuid 10, Nijmegen, The Netherlands; 5 Department of Otolaryngology, Maastricht UMC+, P. Debyelaan 25, Maastricht, The Netherlands

**Keywords:** Cerebellopontine angle, Vestibular schwannoma, Prediction model, Diagnosis, MRI

## Abstract

**Purpose:**

To develop a diagnostic model to identify patients at high risk of a CPA lesion.

**Methods:**

A consecutive cohort of patients with AAD referred by a general practitioner, who underwent their first MRI examination of the CPA between 2005 and 2015 was included. Demographics, symptoms, findings during physical examination, and pure-tone audiometry were used as potential predictors. The presence of a CPA lesion was used as outcome.

**Results:**

We analyzed data of 2,214 patients, detecting 73 CPA lesions in 69 (3.1%) patients. The final model contained eleven variables, namely gender [male] [OR 1.055 (95% CI 0.885–1.905)], sudden onset of hearing loss [OR 0.768 (95% CI 0.318–0.992)], gradual onset of hearing loss [OR 1.069 (95% CI 0.500–1.450)], unilateral tinnitus [OR 0.682 (95% CI 0.374–0.999)], complaints of unilateral aural fullness [OR 1.006 (95% CI 0.783–2.155)], instability [OR 1.006 (95% CI 0.580–2.121)], headache [OR 0.959 (95% CI 0.059–1.090)], facial numbness [OR 2.746 (95% CI 0.548–11.085)], facial nerve dysfunction during physical examination [OR 1.024 (95% CI 0.280–3.702)], and asymmetry in BC at 1 kHz [OR 1.013 (95% CI 1.000–1.027)] and 4 kHz [OR 1.008 (95% CI 1.000–1.026)].

**Conclusion:**

The proposed diagnostic model is a first step in selecting patients with a high risk of a CPA lesion among those with AAD. It needs to be externally validated prior to its implementation in clinical practice.

**Supplementary Information:**

The online version contains supplementary material available at 10.1007/s00405-021-06778-6.

## Introduction

Patients with asymmetrical audiovestibular dysfunction (AAD, asymmetrical hearing loss, asymmetrical tinnitus, dizziness) undergo a magnetic resonance imaging (MRI) examination to screen for lesions in the cerebellopontine angle (CPA). Vestibular schwannoma (VS) is most common, whereas other types of lesions, such as meningiomas or arachnoid cysts, occur less often [1–3].

Because the incidence of CPA lesions in the screening population is low, most MRIs are negative. Optimization of the diagnostic process prior to imaging would ideally reduce the number of MRIs and costs without missing lesions. To illustrate the costs involved, in the Netherlands, an MRI of the CPA costs about €206 (approximately US$245). If all MRIs without VS could be avoided, approximately €293 (or US$347, including price of consultation following MRI) could be saved per patient with AAD [4, 5]. In the Netherlands (17.3 million inhabitants) [6], this could result in potential savings of up to €3.2 million per year [7]. It, therefore, seems worthwhile to investigate new diagnostic strategies to preselect patients with a high risk of a CPA lesion for MRI.

A recent diagnostic meta-analysis did not reveal accurate existing non-imaging screening methods for detection of VS and CPA lesions [8]. Clinicians use information on history, physical examination, and additional tests to generate a differential diagnosis. So far, only one study combined demographics, symptoms and audiometry to create a diagnostic model to select patients with AAD for MRI [9]. However, this study used a case–control design, did not perform an MRI in all controls and it was unclear how cases were diagnosed.

We, therefore, aimed to develop a multivariable diagnostic model for patients with AAD that can be used to identify patients at high risk of a CPA lesion for MRI. Such a model would be a great asset in identifying patients at high risk of a CPA lesion, and could be used to guide doctors and patients in shared decision-making regarding diagnostics and expectations.

## Materials and methods

### Population

The model was developed using a cohort of patients aged ≥ 16 years who visited the otolaryngology department of a university hospital with AAD complaints between January 2005 and February 2015. All consecutive patients referred by a general practitioner and undergoing their first MRI examination of the CPA in our hospital were retrieved from the Radiology database (IMPAX 6.6.1.1527, version 6.6.1.0 2015, AGFA Healthcare N.V., Mortsel, Belgium). Patients referred by an otolaryngologist from another hospital were excluded, because they are usually diagnosed with a CPA lesion prior to referral. Inclusion would result in a higher incidence of CPA lesions compared to the regular screening population. Patients whose complete medical records were missing and patients with incomplete MRI images (e.g., due to claustrophobia) were also excluded.

The study was performed and reported according to the TRIPOD statement [10].

### Outcome

The presence/absence of a CPA lesion within a patient was used as outcome, irrespective of its side and uni- or bilateral presence. CPA lesion diagnosis was based on the original neuroradiologist’s report. The outcome was considered present whenever any type of CPA lesion was suspected based on MRI images as assessed by a neuroradiologist from our institution. Lesions of unknown origin were also considered CPA lesions, because these are usually considered abnormal and included in a (temporary) follow-up policy.

#### Size of CPA lesions

Information was gathered about the size of all VSs and meningiomas at time of diagnosis. In case MRI images were available we determined largest diameter in two directions on axial images: parallel to the internal auditory canal (split in an intra- and extrameatal portion delineated by the petrous bone) and largest diameter parallel to the petrous bone.

### Potential predictors

A list of potential predictors was established based on literature and expert interviews (three otolaryngologists, specialized in neuro-otology/skull base). Information on demographics, symptoms, physical examination, and pure-tone audiometry (PTA) results were collected from the patients’ otolaryngology records using a pre-specified case report form. Potential predictors were selected based on previous studies and expert opinion [8].

#### Demographics

Gender and age were included as potential predictors.

#### Symptoms

Hearing loss was scored asymmetrical whenever a subjective difference was reported between ears (including unilateral complaints). Moreover, we collected data about the onset of hearing loss. Patients were scored to have sudden and/or gradual onset of hearing loss, when it was described as such in at least one ear. Duration of tinnitus was scored as either more or less than two months, the latter also being applied for patients without tinnitus. Complaints of facial numbness and weakness, vertigo, instability and headache were scored as either absent or present. Symptoms were considered absent in case they were not mentioned in the patient record.

#### Physical examination

Facial nerve dysfunction was positive in case any abnormality was described on at least one side.

#### Pure-tone audiometry

PTA examinations performed within six months prior to MRI were included. We ensured blinding (of patients and examiners) by solely including PTAs performed prior to MRI. Data were retrieved from the clinical audiology database system.

We collected hearing thresholds in decibels hearing level (dBHL) of octave frequencies 0.5, 1, 2, 4, and 8 kHz for bone conduction (BC) and air conduction (AC). Absolute asymmetry of BC hearing thresholds between ears was calculated for each frequency. We calculated absolute asymmetry of the low and high Fletcher Index between ears using hearing thresholds in dBHL of octave frequencies 0.5, 1 and 2, and 1, 2 and 4 kHz AC, respectively. All were modeled as continuous variables.

### Data analysis

For 13 of the 20 variables, data were missing (1.7–50.9%) (Table 1). These were imputed using multiple imputation by chained equations procedure using predictive mean matching. The R package mice was used to perform multiple imputation [11]. Missing data were assumed to be missing at random (MAR). The MAR assumption appeared to be valid by visual exploration of missingness [12, 13].

**Table 1:** Overview of patient characteristics and missing data in 2214 included patients

Variable	Descriptives *n* (%)^a^					
	Total *N* = 2214	Missing	CPA lesion *n* = 69	Missing	No CPA lesion *n* = 2145	Missing
Demographics						
Gender (male)	1149 (51.9)	0	41 (59.4)	0	1108 (51.7)	0
Age^b^	58 (16–93)	0	58 (16–86)	0	58 (16–93)	0
Hearing loss						
Asymmetrical	1217 (55.0)	538 (24.3)	46 (66.7)	11 (15.9)	1171 (54.6)	527 (24.6)
Sudden onset^c^	317 (14.3)	1108 (50.0)	8 (11.6)	31 (44.9)	309 (14.4)	1077 (50.2)
Gradual onset^c^	397 (17.9)	1126 (50.9)	20 (29)	31 (44.9)	377 (17.6)	1095 (51)
Unilateral tinnitus	997 (45.0)	0	21 (30.4)	0	976 (45.5)	0
Unilateral aural fullness	277 (12.5)	0	9 (13)	0	268 (12.5)	0
Dizziness						
Vertigo	347 (15.7)	38 (1.7)	9 (13)	0	338 (15.8)	38 (1.8)
Instability	179 (8.1)	38 (1.7)	7 (10.1)	0	172 (8)	38 (1.8)
Headache	77 (3.5)	0	1 (1.4)	0	76 (3.5)	0
Facial complaints						
Facial numbness	29 (1.3)	0	3 (4.3)	0	26 (1.2)	0
Facial weakness	18 (0.8)	0	1 (1.4)	0	17 (0.8)	0
Physical examination						
Facial nerve dysfunction (HB ≥ 2)	20 (0.9)	0	1 (1.4)	0	19 (0.9)	0
PTA asymmetry^d^						
BC 0.5 kHz	10 (0–65)	723 (32.7)	15 (0–55)	25 (36.2)	10 (0–65)	698 (32.5)
BC 1 kHz	10 (0–75)	714 (32.2)	20 (0–70)	25 (36.2)	10 (0–75)	689 (32.1)
BC 2 kHz	10 (0–75)	714 (32.2)	10 (0–65)	25 (36.2)	10 (0–75)	689 (32.1)
BC 4 kHz	10 (0–85)	715 (32.3)	20 (0–75)	25 (36.2)	10 (0–85)	690 (32.2)
BC 8 kHz	0 (0–55)	749 (33.8)	5 (0–40)	28 (40.6)	0 (0–55)	721 (33.6)
High FI	13 (0–117)	521 (23.5)	25 (0–103)	18 (26.1)	13 (0–117)	503 (23.4)
Low FI	13 (0–117)	521 (23.5)	17 (0–103)	18 (26.1)	13 (0–117)	503 (23.4)

*PTA* pure-tone audiometry, *BC* bone conduction, *FI* Fletcher-index

^a^The number of patients and corresponding percentage is reported, unless stated otherwise in the first column

^b^Median years (range)

^c^In at least one ear

^d^Median dB (range)

To determine the number of imputed datasets, an iterative multiple imputation approach, implemented in the R package ‘imi’, was used [14]. Based on this approach 90 imputed datasets were needed.

Model selection after multiple imputation was performed using a penalized logistic regression using least absolute shrinkage and selection operator (LASSO) taking into account the 90 multiple imputed datasets as implemented in the R package ‘MAMI’ [15, 16]. LASSO is considered a suitable method in case of few outcome events (CPA lesions) [17], and it generally results in improved performance and parsimony of a model compared to other shrinkage methods [16, 17].

Thereafter, variable selection was performed and regression coefficients were determined by weighted model averaging, which incorporates uncertainty of model selection in its variance estimates and confidence intervals (CIs) [15, 18]. The regression coefficients’ 95% CIs were obtained using 200 bootstrap samples as described in detail elsewhere [19].

Model performance measures, i.e., calibration intercept, calibration slope, and c-index, were estimated in each imputed dataset, and subsequently pooled using multiple imputation rules (so-called pooled performance strategy) [20]. The model was internally validated using bootstrap resampling for internal validation and estimation of the expected optimism was performed based on Musoro et al. [21].

To easily calculate an individual’s risk of having a VS using the model, a dynamic nomogram was created. The nomogram is available via https://vs-model.shinyapps.io/predictCPA, where more data can be entered and corresponding predictions with their 95% confidence intervals can be calculated.

TRIPOD recommends to evaluate a prediction model’s net benefit [10]. Decision curve analysis (DCA) may help to summarize clinical usefulness of prediction models and support clinicians in decision-making [22, 23]. DCA is a plot of net benefit (NB) against threshold probability.

NB gives the proportion of “net” true positives in the dataset: the observed number of true positives is corrected for the observed proportion of false positives weighted by the odds of the risk threshold, and the result is divided by the sample size. This “net” proportion is equivalent to the proportion of true positives in the absence of false positives (i.e., perfect specificity) [22].

NB is calculated as follows: [24].

$${\text{NB}}\, = \,{\text{True positives}}/N{\text{-false positives}}/N \times p_{t} /\left( {1 - p_{t} } \right)$$

Threshold probability (*p*_*t*_) is defined as the minimum predicted risk of having a CPA lesion at which an otolaryngologist or patient would want an MRI. To represent a variety of preferences, a range of values is displayed [24, 25]. Preferences may vary between individuals. This range of values should be established prior to model reporting. Interviews with otolaryngologists from our center working in the field of CPA lesions revealed that threshold values from 0% (MRI for all patients to find all CPA lesions, regardless of negative MRIs, i.e., the current strategy) to 5% (indicating that one accepts 19 negative MRIs to find 1 CPA lesion in a group of 20 patients) were regarded relevant by them. Although this range is narrow, it provides possibilities to optimize the diagnostic strategy. NB represents the proportion of true positives (diagnosed CPA lesions) in absence of any false positives (specificity of 100%). To obtain standardized NB, the incidence of disease (intercept with *y*-axis) is set to 100%. Using DCA, one can compare the model to a ‘scan all’ (i.e., the current) or ‘scan none’ strategy. The threshold probability is dependent on an individual’s preferences and determines the threshold for MRI referral to screen for CPA lesions.

Furthermore, we plotted the number of MRIs avoided per 1000 patients at risk against the threshold probability, which can be used to assess potential savings (in terms of MRIs) of the model. Additionally, true and false-positive rates were plotted against the threshold probability.

R statistical software version 3.6.0 (The R Foundation for Statistical Computing, Vienna, Austria) with packages ‘imi’, ‘mice’ and ‘MAMI’ were used for data analysis [11, 14, 18].

### Patient and public involvement statement

Representatives of the Dutch patient society for CPA lesions (Stichting Hoormij–NVVS) supported the study protocol. Meetings were organized with representatives to update them on study findings and exchange ideas and comments.

## Results

### Population

We retrieved data of 2,725 adult patients with AAD who had visited our department and had undergone an MRI to screen for CPA lesions. We excluded 511 patients: 103 children, 6 missing patient records, 5 incomplete MRI examinations in which the CPA could not be properly assessed (3 claustrophobia, 2 movement/metal artifacts), and 397 patients were referred by an otolaryngologist from another hospital, resulting in 2,214 inclusions.

#### Diagnostic variables

Mean age at time of consultation was 56 (range 16–93) years and 1149 (51.9%) patients were men. Table 1 displays an overview of patient characteristics and missings per potential diagnostic variable.

#### Outcome

Outcome data were available for all patients. Seventy-three CPA lesions were present in 69 (3.1%) subjects (i.e., 4 bilateral lesions), 42 (57.5%) were located on the right and 31 (42.5%) on the left side. Unilateral VS was found in 46 (2.1%) and bilateral VS in 2 patients (0.1%), 28 and 22 of VSs were found on the right and left side (56% and 44%), respectively. Another 19 (0.9%) patients had a unilateral meningioma (*n* = 7; 0.3%), arachnoid cyst (*n* = 5;0.2%), lipoma (*n* = 1;0.05%), and lesion of unknown origin (*n* = 6;0.3%) in the CPA. One patient had bilateral metastases and one bilateral lesions of unknown origin.

### Final diagnostic model

The final model consisted of eleven variables, namely gender [OR 1.055 (95% CI 0.885–1.905)], sudden onset of hearing loss [OR 0.768 (95% CI 0.318–0.992)], gradual onset of hearing loss [OR 1.069 (95% CI 0.500–1.450)], unilateral tinnitus [OR 0.682 (95% CI 0.374–0.999)], complaints of unilateral aural fullness [OR 1.006 (95% CI 0.783–2.155)], instability [OR 1.006 (95% CI 0.580–2.121)], headache [OR 0.959 (95% CI 0.059–1.090)], facial numbness [OR 2.746 (95% CI 0.548–11.085)], facial nerve dysfunction during physical examination [OR 1.024 (95% CI 0.280–3.702)], and asymmetry in BC at 1 kHz [OR 1.013 (95% CI 1.000–1.027)] and 4 kHz [OR 1.008 (95% CI 1.000–1.026)]. Table 2 displays coefficients and 95% CIs of variables obtained in the final model after internal validation.

**Table 2:** Estimates of the final diagnostic model and 95% confidence intervals

	Coefficient	OR	Lower 95% CI	Upper 95% CI
Intercept	− 3.731			
Gender (male)^a^	0.065	1.055	0.885	1.905
Sudden onset of hearing loss^a^	− 0.325	0.768	0.318	0.992
Gradual onset of hearing loss^a^	0.082	1.069	0.500	1.450
Unilateral tinnitus^a^	− 0.471	0.682	0.374	0.999
Unilateral aural fullness^a^	0.007	1.006	0.783	2.155
Instability^a^	0.007	1.006	0.580	2.121
Headache^a^	− 0.052	0.959	0.059	1.090
Facial numbness^a^	1.242	2.746	0.548	11.085
Facial nerve dysfunction^a^	0.030	1.024	0.280	3.702
Asymmetry in BC at 1 kHz (dB)^b^	0.016	1.013	1.000	1.027
Asymmetry in BC at 4 kHz (dB)^b^	0.010	1.008	1.000	1.026

Probability (P) of having a CPA lesion = 1/ (1 + exp(-lp)), where lp = − 3.73121 + (0.06519 × gender) + (− 0.32472 × sudden onset of hearing loss) + (0.08241 × gradual onset of hearing loss) + (− 0.47109 × unilateral tinnitus) + (0.00738 × unilateral aural fullness) + (0.00738 × instability) + (− 0.05166 × headache) + (1.24230 × facial numbness) + (0.02952 × facial nerve dysfunction) + (0.01599 × asymmetry in BC at 1 kHz) + (0.00984 × asymmetry in BC at 4 kHz)

In the online dynamic nomogram data can easily be entered in the model. It can be found via https://vs-model.shinyapps.io/predictCPA

*OR* Odds Ratio, *CI* confidence interval, *BC* bone conduction, *kHz* kilohertz, *dB* decibel

The probability of having a CPA lesions can be calculated as follows using the regression coefficients presented above

^a^Binary variables: 0 = absent, 1 = present

^b^Continuous variable

The AUC of the model’s ROC curve based on pooled predictions was 0.67 (95% CI 0.59–0.75), indicating acceptable discrimination. The calibration intercept was 0.00 (95% CI − 0.24 to 0.24) and the calibration slope 1.15 (95% CI 0.64–1.66).

**Fig. 1:**
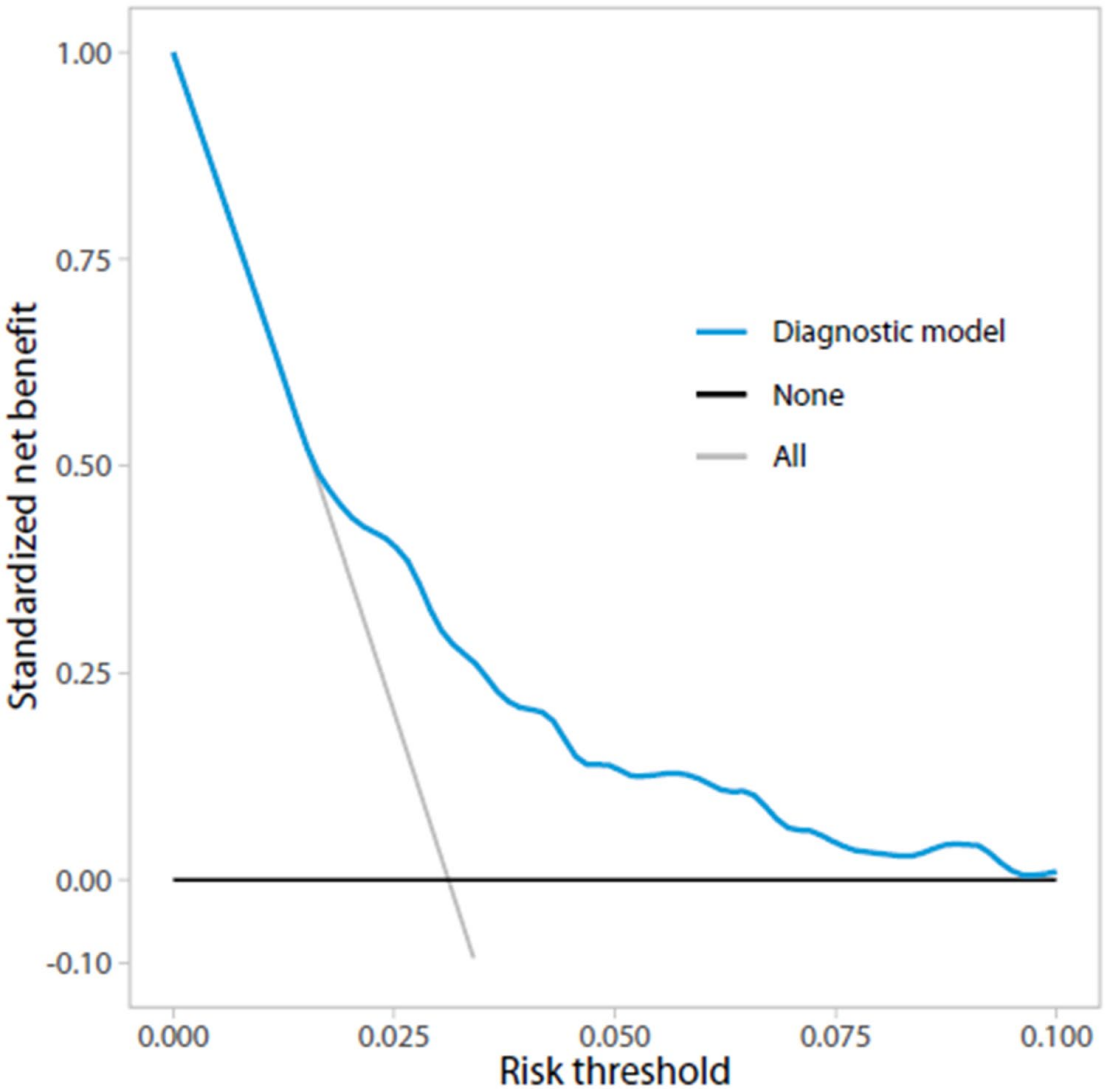
Decision curve analysis: Standardized net benefit curve. *x*-axis = risk threshold; *y*-axis = standardized net benefit; black line = strategy in which no MRIs are acquired, the net benefit is 0; gray line = current strategy, in which all patients have undergone an MRI; blue line = the prediction model

**Fig. 2:**
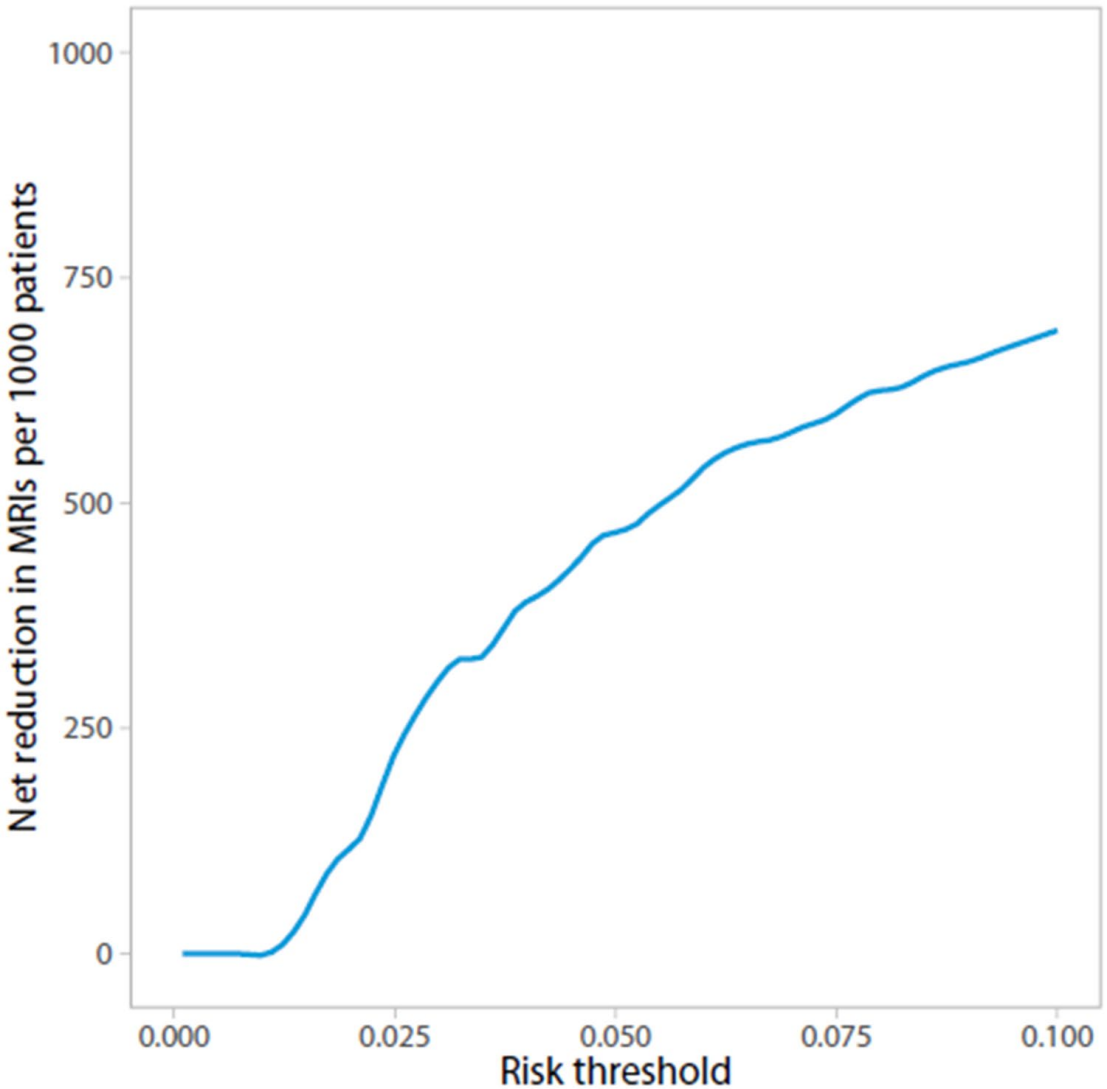
Decision curve analysis: MRIs avoided**.** The x-axis represents the risk threshold, the y-axis the number of MRIs avoided per 1000 patients with AAD

**Fig. 3:**
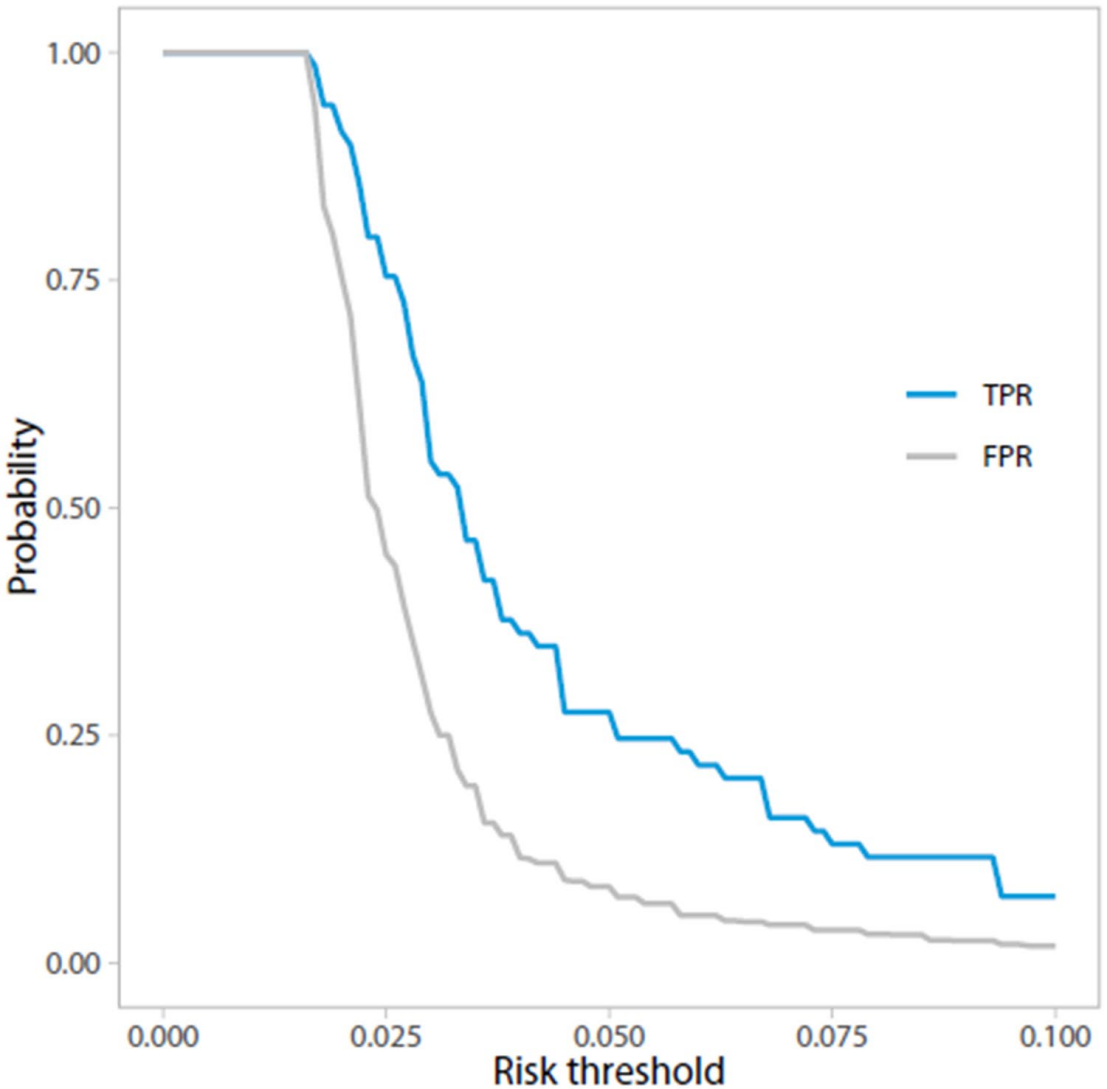
Decision curve analysis: Trsue- and false-positive rate. x-axis = risk threshold;y-axis = probability of patients with true-positive diagnoses (blue line) and false-positive diagnoses (gray line). Decline of blue line indicates that CPA lesions will be missed

The NB curve in Fig. 1 can be used to assess the standardized NB for different threshold probabilities at which one can use the model. The model’s NB is higher than the current ‘scan all’ strategy for risk thresholds > 1.8%. Figure 2 displays the number of MRIs avoided for different threshold probabilities. At a risk threshold of 1.8%, 2.5% and 5%, using the prediction model compared to the current strategy could avoid 1.1%, 22.4% and 44.5% of MRIs, respectively. Figure 3 shows true- and false-positive rates for different threshold probabilities. As the risk threshold increases, the number of patients diagnosed with a CPA lesion decreases.

### Size of CPA lesions

Of all patients with a VS, MRI images were not available for two patients. Of the patients with a unilateral VS, three had an intracochlear VS of which measurements were not included in our analyses. Of 45 remaining VSs (including two patients with bilateral VSs), 27 patients had a purely intrameatal localization of their VS. Mean size of the intrameatal portion parallel to the internal auditory canal was 6.1 mm (median 5 mm, range 0–14 mm). The mean extrameatal portion parallel to the internal auditory canal was 4.3 mm (median 0 mm, range 0–25 mm) and the mean size parallel to the petrous bone was 7.9 mm (median 5 mm, range 1–30 mm). Among the seven patients with a meningioma, mean intrameatal size was 2.1 mm (median 0, range 0–11 mm). The mean extrameatal portion parallel to the internal auditory canal was 12.3 mm (median 14 mm, range 4–21 mm) and parallel to the petrous bone mean size was 15.9 mm (median 13, range 5–37 mm).

## Discussion

We developed a clinical prediction model to identify those with high risk of a CPA lesion in AAD patients. This model contains eleven variables, i.e., gender, sudden onset of hearing loss, gradual onset of hearing loss, unilateral tinnitus, unilateral aural fullness, instability, headache, facial numbness, facial nerve dysfunction and asymmetry in BC at 1 and 4 kHz. Presented decision curves can be used to compare the clinical value of the prediction model with the current ‘scan all’, and a ‘scan none’ strategy. The model should be externally validated prior to its use in clinical practice.

To our knowledge, this is the first cohort study creating a prediction model for patients with AAD. We included primary referrals, to ensure our study population represented a standard screening population instead of that of a tertiary referral center (where diagnoses are usually established beforehand). All patients underwent MRI, so outcome data were available for all patients. Moreover, input variables for the diagnostic model can be acquired easily through history taking, physical examination and PTA. Patients and examiners are ideally blinded for the outcome when determining values of potential diagnostic variables. We ensured blinding by solely including PTAs performed prior to MRI, and used a timeframe of 6 months to ensure that PTAs were representative for hearing levels at the time of MRI.

Some potential limitations should also be discussed. First, data were collected from patients’ records. Not all required information could be derived, resulting in missing values. We used multiple imputation, which is recommended compared to complete case analysis [12, 26]. Whenever complaints were not reported in the patient record, they were assumed to be absent. Possibly, some complaints were not reported by the otolaryngologist, or not mentioned by a patient. We assume however, that most complaints included in our analyses are usually registered, because they are common in patients with a suspected CPA lesion. Second, criteria for MRI referral are known to vary [27]. Our local protocol prescribes an MRI for asymmetries of ≥ 10 dB on three consecutive frequencies, unilateral constant tinnitus ≥ 3 months, unilateral decreased/absent vestibular function. However, our national protocol on sensorineural hearing loss does not specify the amount or frequencies of asymmetry required for MRI referral. The group of included patients might therefore be heterogenous considering their hearing loss, but comprise patients that currently would undergo MRI and are therefore representative for current practice. Third, ideally one would include all potential predictors mentioned in literature. Due to the retrospective nature of our study, we had to rely on available data. For example, the use of speech audiometry and its correlation with PTA would be interesting to investigate*.* Fourth, it should be noted that coefficients of most predictors are close to zero, partially due to LASSO shrinkage, i.e., their individual contribution to each prediction is rather small. Consequently, external validation may pose a challenge.

New screening methods to select patients with AAD for MRI will undoubtedly result in false negative results. Currently, it is a challenge to assess consequences of missed CPA lesions resulting from this diagnostic model, for both patients (e.g., quality of life and functional outcomes) and society (i.e., costs). The majority of patients with a VS are obtained in a ‘wait-and-scan’ policy. A large proportion of VSs does not grow and thus remains untreated for years [28–30]. One could question the need of diagnosing CPA lesions for which therapeutic consequences are lacking.

We believe that a next step in optimizing the diagnostic process of CPA lesions would be to focus on diagnosing those lesions requiring treatment (i.e., larger/growing lesions).

Tinnitus is often considered an indication for an MRI [31]. The coefficient of ‘unilateral tinnitus’, however, turned out to be negative in our prediction model, which seems contradictory with clinical practice. Positive predictive value of unilateral tinnitus was previously shown to be low [32, 33], which also results from our data: 17 and 21 out of 997 patients (1.7% and 2.1%) with unilateral tinnitus are diagnosed with a VS and CPA lesion, respectively. These numbers are lower than the incidence of CPA lesions in our data, which explains the negative coefficient. Moreover, prior to MRI, complaints of uniltaral tinnitus cannot yet be linked to the ear affected by a lesion [34].

Although this diagnostic model is a first step in selecting patients at high risk of a CPA lesion for MRI, its diagnostic accuracy would preferably be improved prior to its clinical use. Ideally, it would be possible to eliminate misdiagnoses (particularly false negatives), provide a safety net for false negatively diagnosed patients, or safely state that consequences of false negative diagnoses are negligible, before this diagnostic model can be safely used. Moreover, the TRIPOD statement highly recommends external validation [10]. Preferably, this is done using prospectively collected data. DCA was used to compare the model’s NB to a ‘scan all’ (i.e., the current) and ‘scan none’ strategy. DCA showed that recommending MRI if the predicted risk of a CPA lesion is 1.8% or more results in a higher net benefit compared to scanning all patients. In other words, if the predicted risk of a CPA lesion is 1.8% or more, using the model to determine whether patients should have an MRI would lead to improved clinical decisions compared to scanning all patients. Eventually, an impact study is needed to evaluate cost-effectiveness; costs and effects of applying the diagnostic model in clinical practice need to be compared to the current diagnostic strategy in which an MRI is acquired in all patients with AAD.^35^ This diagnostic model can potentially help focus the otolaryngologist’s attention in history taking and requesting additional tests, but also may help patients decide whether they want to undergo an MRI.

## Conclusion

The proposed diagnostic model, containing eleven variables that can easily be assessed in every otolaryngology practice using history taking, physical examination and PTA, is a first step in identifying patients with a high risk of a CPA lesion among those with AAD. Following external validation, clinicians may use the model to support their clinical decisions (MRI for all or MRI based on the predicted risk of a CPA lesion) and may use it in shared decision-making.

## Supplementary Information

Below is the link to the electronic supplementary material.

Supplementary file1 (R 10 KB)

Supplementary file2 (R 8 KB)

## Data Availability

The code is available as supplemental resource.

## Declarations

### Availability of data and material

The datasets used and/or analyzed during the current study are available from the corresponding author on reasonable request and approval by the local privacy officer.
